# A Porcine Epidemic Diarrhea Virus Outbreak in One Geographic Region of the United States: Descriptive Epidemiology and Investigation of the Possibility of Airborne Virus Spread

**DOI:** 10.1371/journal.pone.0144818

**Published:** 2015-12-28

**Authors:** Andrea Beam, Dane Goede, Andrew Fox, Mary Jane McCool, Goldlin Wall, Charles Haley, Robert Morrison

**Affiliations:** 1 USDA Animal and Plant Health Inspection Service, Veterinary Services, Science, Technology, and Analysis Services, Center for Epidemiology and Animal Health, Fort Collins, Colorado, United States of America; 2 Department of Veterinary Population Medicine, College of Veterinary Medicine, University of Minnesota, Saint Paul, Minnesota, United States of America; Sun Yat-sen University, CHINA

## Abstract

This study describes a spring 2013 outbreak of porcine epidemic diarrhea virus (PEDv), using data from 222 swine sites in 14 counties area in 4 contiguous states in the United States. During the outbreak, the premises-level incidence of PEDv was 40.5 percent (90/222 sites). One of the three companies from which data were collected had a lower incidence (19.5 percent) than the other two companies (41.1 and 47.2 percent). Sow sites had the highest incidence of PEDv during the outbreak (80.0 percent). Spatial analysis showed that PEDv was clustered rather than randomly distributed, which suggested that sites near a positive site had increased risk of acquiring PEDv infection. Meteorological data were used to investigate the hypothesis that PEDv was spread by air. If airborne dissemination played a role in this outbreak, we would expect the direction of disease spread to correlate with the predominant wind direction. Two methods were used to determine the direction of disease spread—linear direction mean analysis in ArcGIS and the direction test in ClusterSeer. The former method indicated PEDv spread was south to slightly southwest, and the latter indicated spread was to the southeast. The predominant wind direction during the month of the outbreak was toward the south, with some southeast and southwest winds; the strongest wind gusts were toward the southwest. These findings support the hypothesis that PEDv was spread by air. The results, however, should be interpreted cautiously because we did not have information on direct and indirect contacts between sites, such as movement of trucks, feed, pigs or people. These types of contacts should be evaluated before pathogen spread is attributed to airborne mechanisms. Although this study did not provide a definitive assessment of airborne spread of PEDv, we believe the findings justify additional research to investigate this potential mechanism of transmission.

## Introduction

Since it was first recognized in the United States in May 2013, the porcine epidemic diarrhea virus (PEDv) has spread to 30 states [1]. It was estimated the disease has killed more than 10 percent of U.S. pigs and might cause production losses in 2014 of up to 7 percent [2].

The source of the virus in the United States is not known. The virus appeared at about the same time at four separate operations and the sequences were similar, which suggests a common source [3]. While the U.S. PEDv strain has high identity (>99.0%) to some of the 2011–2012 China strains [3], it is not known how the virus arrived in the United States. Similarly, while some transmission mechanisms have been determined (e.g., trucks contaminated with fecal material), other mechanisms are suspected. As the number of affected operations in the United States continues to increase, it is imperative to learn more about how the virus is spread. This study evaluated one possible mechanism of spread: airborne transmission.

Porcine epidemic diarrhea virus (PEDv) is an enveloped positive polar single-stranded RNA group 1 coronavirus most closely related to human coronavirus (HCoV)-229E and transmissible gastroenteritis virus (TGEV) of swine [4]. PEDv is highly contagious, with an incubation period of three to four days [5]. Fecal-oral transmission is believed to be the main mode of transmission. Clinical signs of PEDv may vary widely and are dependent on age of pig, previous exposure, and the immunological status of the farm. The clinical presentation of PEDv may be indistinguishable from that of TGEV. The primary clinical finding is watery feces, which may be flocculent and fetid, lasting 3 to 4 days in all ages of swine. Swine might vomit. Dehydration and metabolic acidosis may be secondary signs. PEDv may spread more slowly than TGEV. If swine recover, it is usually after 7 to 10 days [6]. Morbidity can approach 100 percent in all ages of susceptible swine [6]. In suckling pigs, mortality commonly reaches 50 to 80 percent, but declines to 1 to 3 percent in grower pigs [6].

PEDv has occurred in European and Asian countries for several decades. In China, vaccination was widely used in the swine industry and PEDv prevalence was relatively low. However, in late 2010 China began experiencing an epidemic that was attributed to newer strains of PEDv that could circumvent immunity developed from current vaccines [7].

Mechanisms by which PEDv spreads are not well known. Virus spread is thought to occur primarily through infected pigs, along with some indirect spread through contaminated fomites and transport trucks. A University of Illinois study conducted shortly after the outbreak began in the U.S. evaluated the role that slaughter facilities and other livestock collection points could play in PEDv transmission. Findings indicated that of trailers that were not contaminated at arrival, 11% were contaminated during unloading [8].

Other animals appear to be capable of transmitting virus. A study in Korea suggests that cats might play a role in transmission of PEDv on swine farms [9]. PEDv was detected in 4.2% of cats (1 of 24 in the study); the virus was found in the tonsils. Experience in the U.S. suggests that PED virus spread could be associated with bird traffic, especially in combination with feed-delivery practices in which bulk bins are left open to reduce driver foot traffic and/or allow feed-delivery equipment to come into contact with bins [10].

Some studies have pointed to the possible role of airborne transmission. In the U.S., airborne viable PED virus was detected in an isolation room with infected pigs and PEDv genetic material (not viable) was found by PCR in air up to 10 miles downwind from an infected site [11].

Given the potential for airborne transmission of PEDv, one objective of this report was to use geospatial methods and meteorological data to analyze a PEDv outbreak in one geographic region of the United States to investigate the hypothesis that PEDv was spread by air. An additional objective was to describe the outbreak epidemiologically.

## Methodology

### Data sources

Three swine companies provided data for all of their production sites in a 14-county area in 4 contiguous states, a total of 242 sites. The three participating companies did not account for all swine production sites in the 14-county area. The names of participating states, counties, and companies, and depictions of exact geographic coordinates, are not provided in this manuscript to protect confidentiality of the participants. For each of the 242 sites, data were available on production type (finisher, nursery, sow, other type); premises identification number; PEDv infection status; and, for sites with PEDv, the accession date for PEDv sample submission to a veterinary diagnostic laboratory. The PEDv-infection-status variable had three levels: negative, positive, and positive placed. The positive-placed category represented sites that received pigs from a known PEDv-positive site.

With permission from the participating producers and state veterinarians, geographic coordinates for sites were obtained from the USDA national premises identification repository. Coordinates were verified using Google Maps. Twenty sites were excluded from further study because their available geo-coordinates were inaccurate, leading to a total of 222 swine sites for the analysis. In addition, wind data from local weather stations were collected from the National Oceanic and Atmospheric Administration [12] and were processed and analyzed. All data received or linked with the original data are subject to the Confidential Information Protection and Statistical Efficiency Act of 2002, Public Law 107–347, December 17, 2002, Title V, Confidential Information Protection and Statistical Efficiency.

The data providers and the authors have signed a legal agreement so that the data is protected to the extent allowable by Federal law and regulation and remains the property of the data provider(s).

### Data analysis

Data tabulations and an epidemic curve were created using Microsoft® Excel® 2010 (14.0.7109.5000), and tabulations for PEDv-positive sites were combined with PEDv-positive placed sites. Cases were defined as sites that were PEDv-positive or PEDv positive-placed. Weekly PEDv incidence was calculated by dividing the number of new cases in a given week by the number of uninfected sites (population at risk) at the beginning of the week. Incidence by company was compared using a chi-square test in SAS® 9.3.

Data were then imported into ArcGIS for analysis. A hotspot analysis (Getis-Ord Gi*) was performed to identify statistically significant geographic clusters of PEDv cases. The ArcGIS “Optimized Hotspot” tool was used for this analysis. The tool evaluates the characteristics of the outbreak data by running a Global Moran’s I test to identify distances in which clustering appears within the dataset. A neighborhood search radius of 11.5 miles was selected for this cluster analysis based on the results of the Global Moran’s I statistic. A hotspot surface was then generated using resulting z-scores from the analysis as input to the inverse distance weighting interpolation tool. The resulting surface shows geographic areas with significant clusters of positive sites in red and areas without significant clustering of positive sites in blue. A directional ellipse tool in ArcGIS was also used to describe the overall directional orientation of PEDv positives from week to week. This tool uses the standard deviation of the x and y coordinates from the mean center (of weekly incident cases) to define the axes of the ellipse. The resulting weekly ellipses show the distribution and orientation of PEDv cases on a weekly scale.

**Fig 1:**
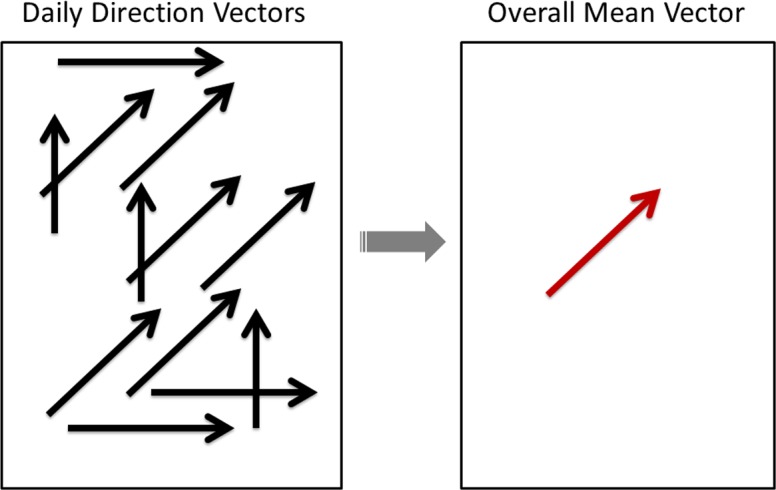
Graphic depiction of ArcGIS linear directional mean function. The ArcGIS linear directional mean function converts the mean daily vectors for direction and distance of virus spread (left) into a single vector that indicates the mean direction and mean distance of virus spread over time.

Direction of PEDv disease spread was analyzed and qualitatively compared with the wind data. Two methods were used to describe the direction of disease spread during the outbreak: the linear directional-mean function in ArcGIS and the direction method in ClusterSeer. All PEDv cases (including positive-placed sites) were included in the analysis to determine the direction of pathogen spread. For the first method, general direction was determined by first calculating the mean geographic center of incident cases for each day of the outbreak. The mean geographic centers were then used to create a vector indicating the direction and distance of PEDv spread from one day to the next for each day of the outbreak. These mean daily vectors were then used as inputs to the linear directional-mean function in ArcGIS. The linear directional-mean function outputs a single vector indicating the mean direction and mean distance of PEDv spread for all cases (Fig 1). The function also outputs numeric values indicating the average distance of pathogen spread, average direction of spread, and a directional variance value indicating how much the daily vectors deviate from the average direction.

The direction method [13] in ClusterSeer was also used to evaluate the direction of disease spread. This method tests for a space-time interaction and calculates the average direction of the spread. A relative model was used, which connects each case to all subsequent cases and is appropriate for directional processes that operate on a relatively lengthy time scale. The null hypothesis is that cases following (in a temporal sense) a given case are located in a random direction. The alternative hypothesis is that subsequent cases are located in a specific direction. We selected the relative model for two reasons. First, case sites likely shed virus for several weeks. Second, this outbreak lasted only 31 days. Therefore, we believe that each case site had the potential to infect subsequent sites throughout the duration of the outbreak. ClusterSeer provides the following results: a significance test for the above hypothesis, the average direction of disease spread, and a measure of the variance in the angles between connected cases.

Finally, the positive-placed sites were excluded from analysis, and the linear directional mean methodology was repeated to ascertain if the direction of disease spread changed when positive-placed sites were excluded. Statistical significance was considered to be p<0.05.

## Results

### Outbreak description

**Fig 2:**
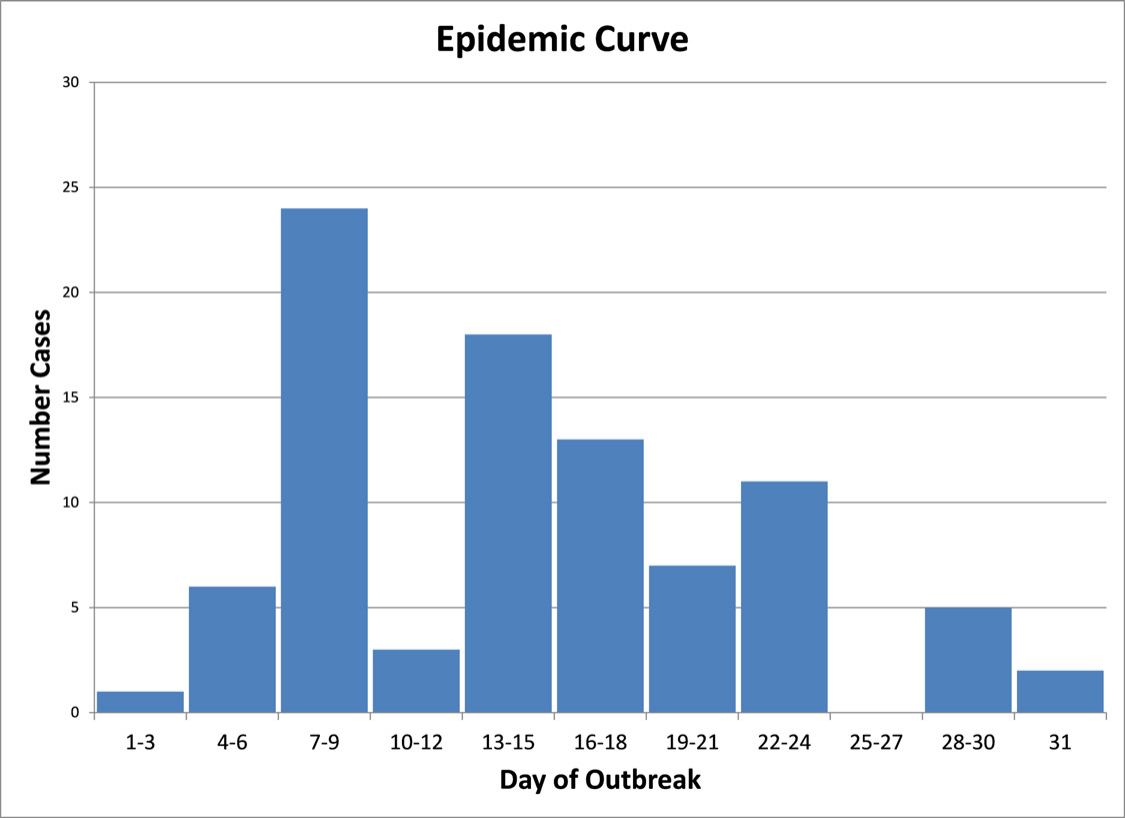
Epidemic curve for a Spring 2013 porcine epidemic diarrhea virus (PEDv) outbreak in 4 contiguous U.S. States. Several peaks occurred, as expected with a propagated outbreak.

In total, 222 sites were included in the analysis. The outbreak at these sites occurred in late spring 2013 and lasted 31 days. During the outbreak, 40.5 percent (90/222 sites) became PEDv positive. One of the companies had a lower site-level PEDv incidence (19.5 percent) than the other two (47.2 and 41.1 percent) [Table 1]. Sow sites had the highest incidence of PEDv during the outbreak (80.0 percent). Of the 90 positive sites, there were 4 positive placed premises, and they were of the nursery and finisher site type. [Table 2]. The epidemic curve for the outbreak suggests that several peaks occurred, as expected with a propagated outbreak (Fig 2).

**Table 1:** Porcine epidemic diarrhea virus (PEDv) site-level incidence during a Spring 2013 outbreak in 4 contiguous U.S. States, by Swine Company.

Company	Percent sites PEDv positive
A	19.5^a^
B	47.2^b^
C	41.1^b^
Total	40.5

Different superscripts indicate a statistically significant difference (p<0.05).

**Table 2:** Porcine epidemic diarrhea virus (PEDv) site-level status during a Spring 2013 outbreak in 4 contiguous U.S. States, by production type.

Production type	Number positive sites	Total	Percent sites PEDv positive
Finisher	48	126	38.1
Nursery	4	45	8.9
Sow	32	40	80.0
Other or unknown type	6	11	54.5
Total	90	222	40.5

### Hotspot analyses (Getis-Ord Gi*) and Weekly directional ellipses

**Fig 3:**
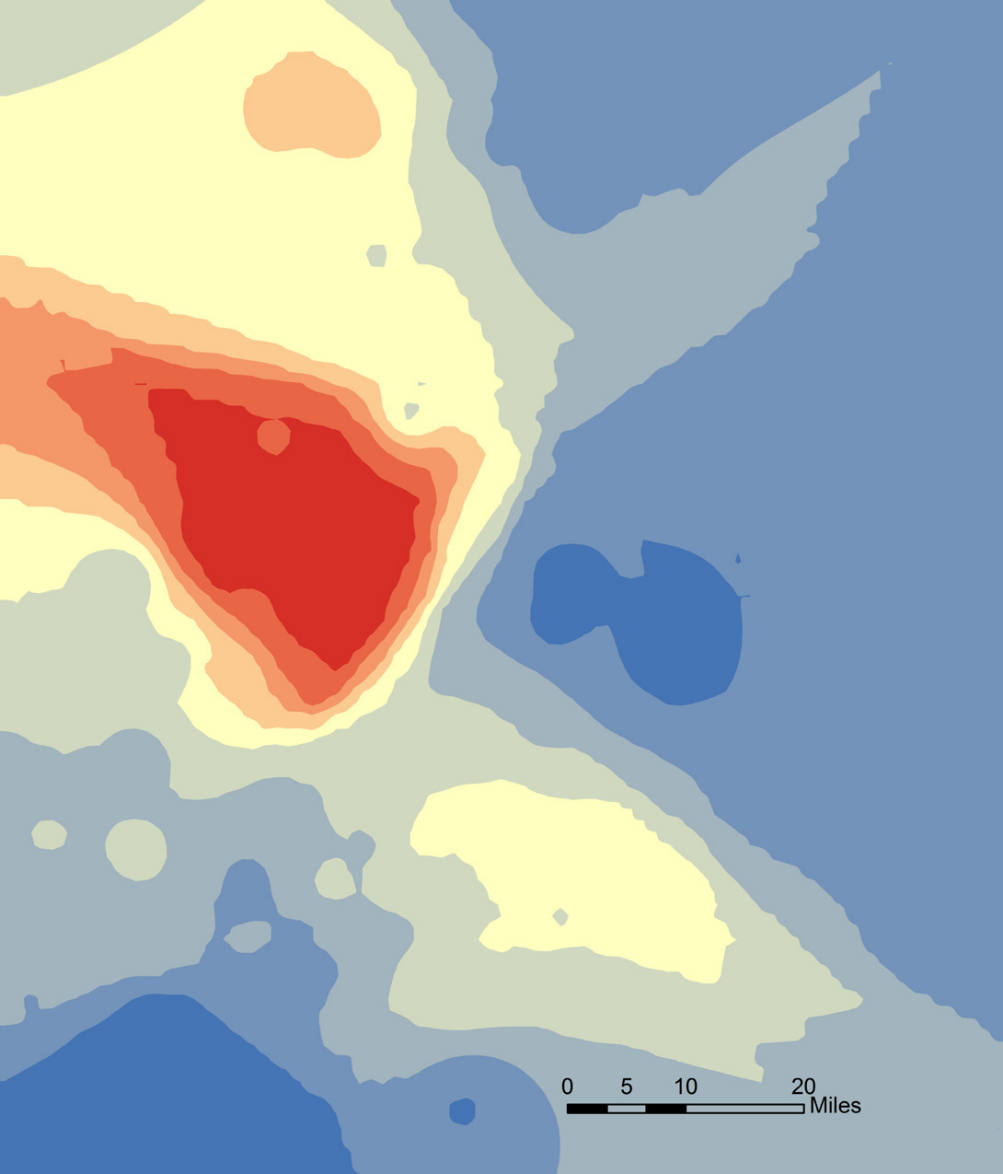
Hotspot cluster analysis of a Spring 2013 porcine epidemic diarrhea virus (PEDv) outbreak in 4 contiguous U.S. States. The red shades show the core cluster, which measured about 33 miles in the north-south direction and 41 miles in the west-east direction.

A statistically significant cluster of positive cases was present in this outbreak. The core cluster measured about 33 miles in the north-south direction and 41 miles in the west-east direction (Fig 3).

**Fig 4:**
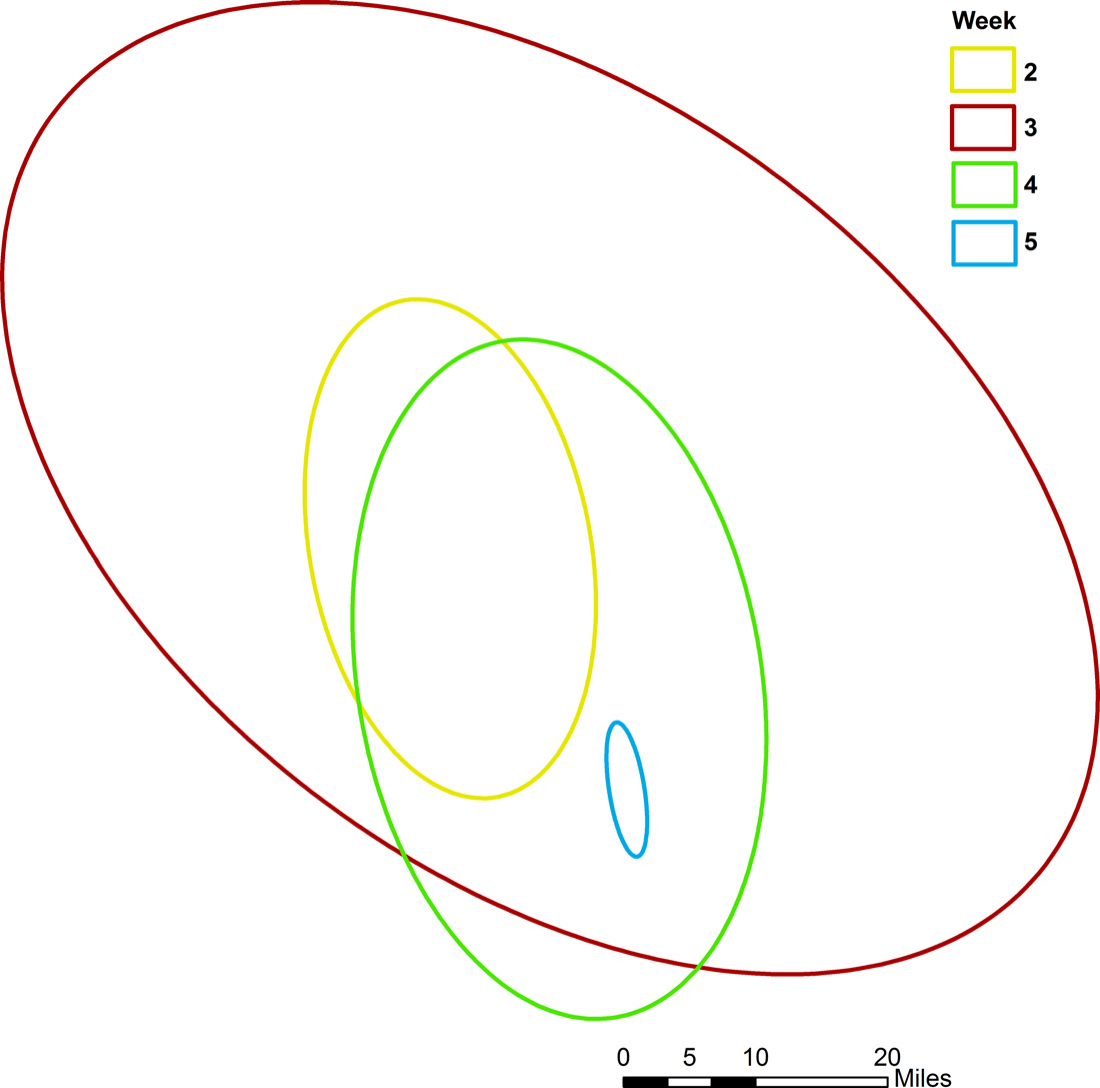
Weekly directional ellipses showing an overall north-south orientation of virus spread. No ellipse was generated for the first week because only one positive was reported that week. During the third week, an outlying positive in the southeastern extent of the outbreak caused a northwest to southeast orientation.

The directional ellipse tool revealed an overall north-south orientation with this outbreak. Note that only one positive case was reported the first week so no ellipse was generated (Fig 4). A slight northwest to southeast orientation appears in the third week due to an outlying positive in the southeastern extent of the outbreak.

### Correlation between wind direction and pathogen spread

**Fig 5:**
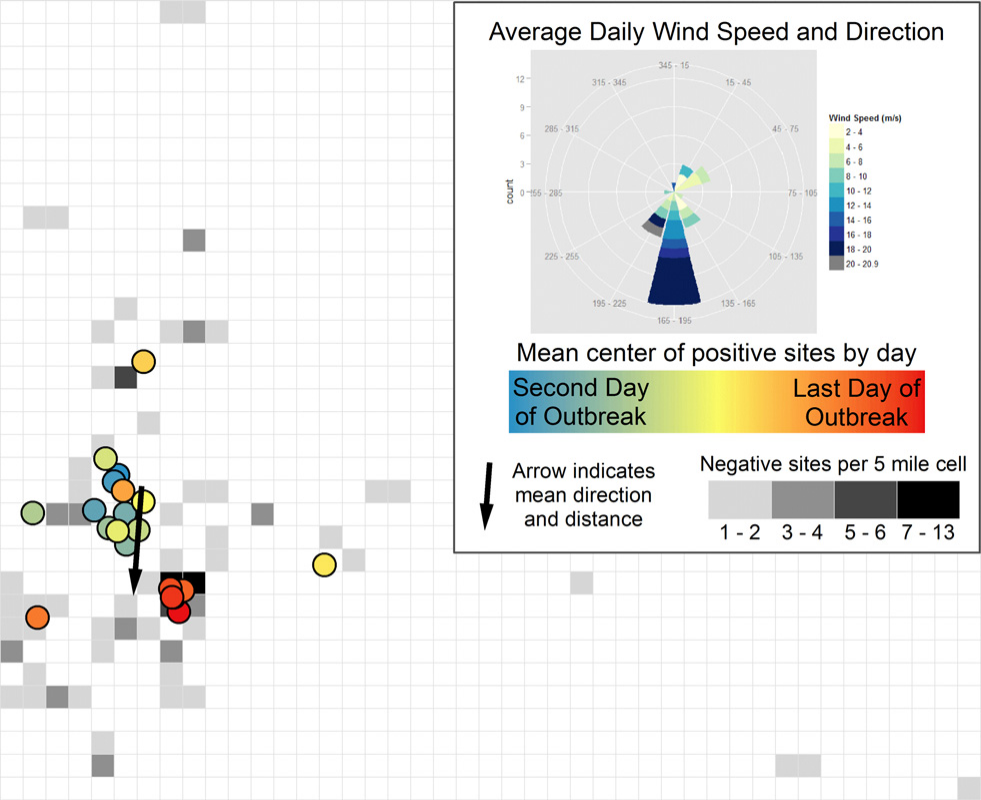
Direction of wind and disease spread during a Spring 2013 porcine epidemic diarrhea virus (PEDv) outbreak. The box on the upper right side of the figure summarizes wind data for the entire outbreak period, and the left side of the figure summarizes results from the linear directional-mean analysis for the direction of disease spread. On the left side, each colored circle represents the mean geographic center for all incident cases on a given day. The blue circles are earlier dates in the outbreak, and the red circles are later dates in the outbreak. The gray squares show locations of sites that remained negative for PEDv; each square measures 5 by 5 miles. Squares with a darker gray color have more negative sites in the area. The black arrow on the left side is the overall direction of disease spread from the linear directional-mean analysis. The mean direction of spread during the entire outbreak was south at 184.2 degrees (measured clockwise from due north), and the mean distance of spread was 19.3 miles.

PEDv spread and the predominant wind direction during the outbreak period generally aligned in a southerly direction (Fig 5). The circular variance from the linear directional-mean analysis was large (0.81), however, indicating that the daily PEDv vectors used as inputs deviated from the directional mean output. This result indicates variability in the directions of the input vectors. Nonetheless, the average direction of PEDv spread with this methodology was south to slightly southwest, and when positive placed sites were excluded from the linear directional mean analysis, PEDv spread direction was still south.

Based on the directional test in ClusterSeer, subsequent cases typically occurred in the southeasterly direction (126.6 degrees^1^ on average) to previous cases. The test statistic indicated this directional pattern was statistically significant (p = 0.001; we could reject the null hypothesis that disease spread was in a random direction). The variance indicated there was considerable variation in the angle directions from one case to subsequent cases (“concentration” = 0.20).

## Discussion

This study described an outbreak of PEDv that occurred in four contiguous states over a 31-day period in spring 2013. The description of this outbreak may be useful in understanding factors that influence the spread of PEDv. Several spatial methods were used to describe the outbreak. The hotspot analysis showed that, overall, the disease was clustered rather than randomly distributed. This result suggested that sites in proximity to a positive site were at increased risk of acquiring PEDv. One limitation of the data in this study was that negative sites were not tested to verify their negative status. Because clinical signs of PEDv are more noticeable in sow farms, it is possible that clinical signs were overlooked on some farms with growing pigs, which would result in them being misclassified as PEDv negative.

**Fig 6:**
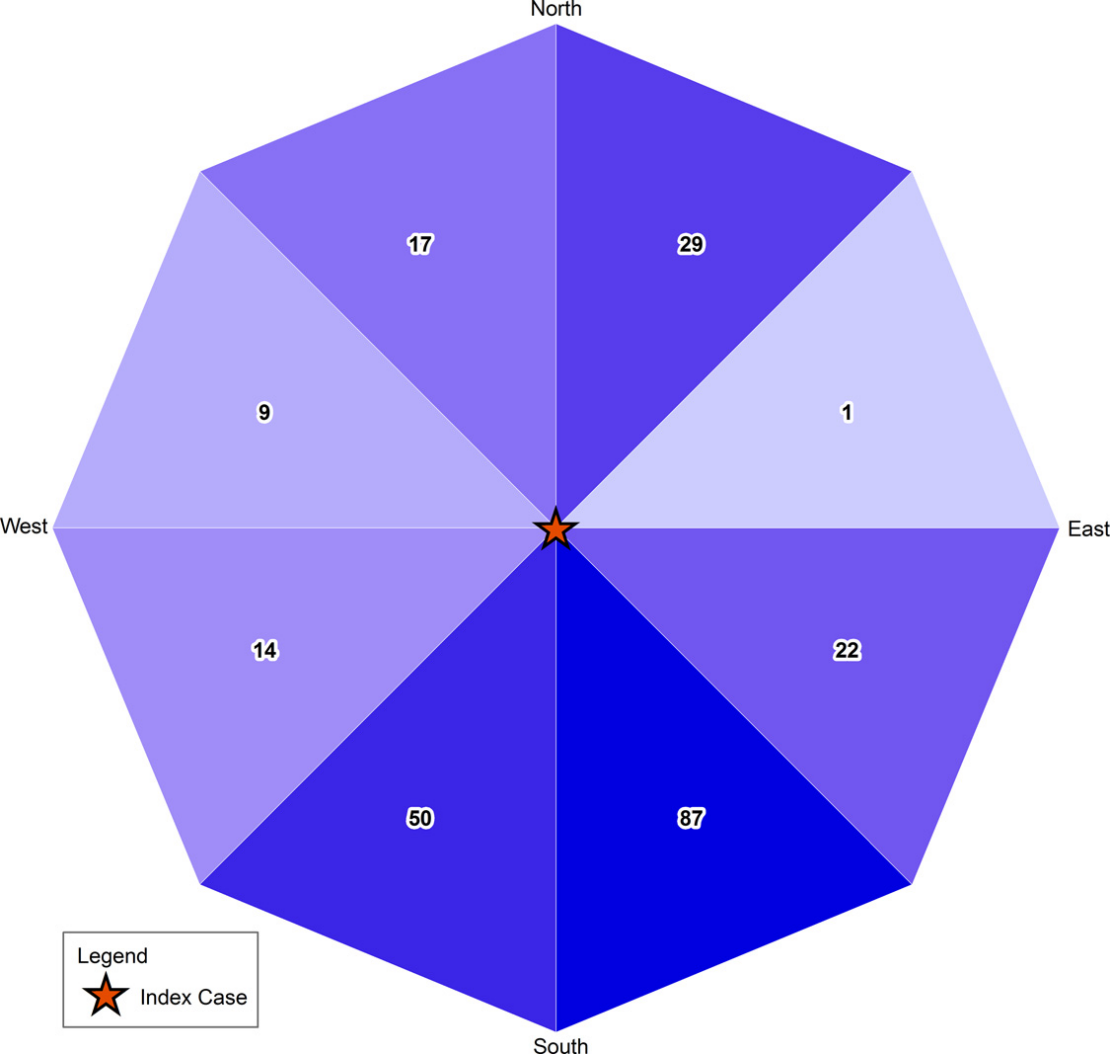
Geographic distribution of swine farms in the study in relation to the first case during a Spring 2013 outbreak of porcine epidemic diarrhea virus (PEDv). More than three-fourths of sites are south of the index case (indicated by red star), with 60 percent in the path of primary wind direction.

We also examined the possibility of airborne spread of PEDv. If airborne dissemination of PEDv played a role in this outbreak, we would expect the direction of disease spread to be correlated with the predominant wind direction. We used two methods to determine the direction of disease spread, and both had similar results: one indicated spread was south to slightly southwest, and the other indicated spread was to the southeast. The slight difference in results is attributable to the different underlying methodologies. The predominant wind direction during the outbreak was toward the south with some southeast and southwest winds; the strongest wind gusts were toward the southwest. Therefore, our results provide support to the hypothesis of airborne PEDv spread, since the direction of disease spread correlated qualitatively with wind direction. The results, however, should be interpreted cautiously because our analysis has a number of limitations. First, we did not have information on direct and indirect contacts among sites, such as movement of trucks, feed, pigs, people, and equipment. These contacts should be considered before pathogen spread is attributed to airborne mechanisms. For example, if truck movement predominately occurs in a southern direction, this could explain the southern movement of PEDv. In addition, swine farm density in the area needs to be considered because unequal distribution of swine farms could influence the observed direction of disease spread. To analyze the distribution of swine farms in the area, we calculated the number of swine farms at risk (uninfected swine sites in our dataset) in the eight primary compass directions relative to the index (first) case. The results of this analysis show that 76 percent of the population at risk was located south of the index case (Fig 6), which could also be a factor in the apparent southern movement of PEDv. Furthermore, we did not consider other meteorological variables such as temperature and humidity, which may also play a role in airborne pathogen transmission.

Finally, several limitations relate to our use of meteorological data, specifically wind information. Wind can generate complex air movement patterns due to changes throughout the day and week. Therefore, we can look only at general trends. Also, wind can vary greatly over the course of a day or week. This analysis considers the predominant wind direction. It is possible, however, that strong gusts of wind occurred in other directions. These gusts could have spread virus in directions other than the predominant wind direction. Furthermore, to more completely describe the influence of wind, we need more information about how far a virus travels at various wind speeds and about what particle sizes (e.g., dust) could carry PEDv. Without knowing the transport mechanism in the air, it is difficult to say what influences wind direction and wind speed could have on the spread of PEDv among sites.

Although our findings are not a definitive assessment of airborne spread of PEDv, we believe they justify additional research on the possibility of airborne spread. A similar analysis of wind direction could be conducted on data from outbreaks in other geographic regions in the United States. Additional studies should include information on the movements of trucks and pigs, as well as other movements that could cause virus spread, if possible. In addition, testing of more air samples with PCR and bioassay are recommended. Sequencing of isolates could more accurately track movement of specific virus in future research and was another limitation of this study. Studies have shown that infectious virus can persist in fecal slurry for at least 28 days at 4°C and for at least 14 days but less than 28 days at room temperature [14], and it could be important to determine if slurry could be aerosolized or carried aloft by the wind as small fecal clumps that could protect the virus. Additionally, although it has been shown that infectious virus survives for less than 2 weeks on dry feed mixture at room temperature [14], it would be helpful to determine how long the virus can survive on dry matter, which could be easily borne on the wind. Flies and other insects may also play a role in disease transmission, and insects may be affected by wind patterns. Therefore, research is also needed to evaluate whether PEDv on or in flies can infect pigs and, if so, whether the flies are biological or mechanical vectors.
